# Growing up with Radicalized Parents: The Experiences of Dutch Children of NSB and SS members During and After World War II

**DOI:** 10.1007/s40653-024-00656-z

**Published:** 2024-09-13

**Authors:** Mattie van der Molen, Brenda Jansen, Bertjan Doosje, Hans te Brake, Conny van Doorn, Marjan van der Zee, Arnold van Emmerik

**Affiliations:** 1 Department of Psychology, Faculty of Social and Behavioural Sciences, University of Amsterdam, Amsterdam, Netherlands; 2 ARQ Centre of Expertise for the Impact of Disasters and Crises, National Psychotrauma Centre Diemen, Diemen, Netherlands; 3 Dutch Child Care and Protection Board, The Hague, Netherlands

**Keywords:** Children, Growing up, Extremist environment, Radicalisation, World war II, Minor returnees, Caliphate

## Abstract

The primary aim of this study was to investigate the experiences of Dutch children whose parents joined the SS or NSB (a political party that collaborated with Nazi Germany) during World War II, linked to their childhood, adulthood or both. As a secondary aim, it explored the recommendations of these –now elderly- children of NSB and SS members for the (re)integration of minor returnees from the caliphate, who also grew up in a war situation with radicalized parents and have to deal with considerable prejudice and different norms and values upon their (re)integration into Dutch society. The first three research questions are related to the primary aim of this study and focus on 1) the participants' experiences throughout childhood and adulthood, 2) the implications of these experiences for their well-being throughout childhood and adulthood, and 3) their coping responses in response to these experiences throughout childhood and adulthood. The fourth research question is related to the secondary aim of this study and concerns 4) participants' recommendations for the succesful (re)integration of current minor returnees. Semi-structured interviews were conducted with 17 Dutch -now elderly- children of NSB or SS members during World War II. Interview questions were based on recurring topics in the literature, a codebook was generated, and the verbatim transcribed interviews were scored using Atlas.ti 8. Frequently reported codes are described and discussed in relation to each other. Participants reported social exclusion, being separated from their parents, and family secrecy as frequent experiences, which had severe implications for their identity development, self-esteem, and family relationships. Their coping responses included reappraisal, active (e.g., searching information) and avoidant (e.g., merel socially desirable) coping behaviour. Participants’ main recommendations for minor returnees from the caliphate included transparency within families about the past, a focus on social (re)integration, and respecting minor returnees’ loyalty to their parents. The participants' childhood experiences had serious effects on their well-being, starting in childhood and often lasting well into adulthood. Many participants sought professional support throughout their lives, but eventually found recognition only after sharing their experiences in a self-help group for Dutch children of NSB and SS members. We conclude that the insights gained from participants’experiences and their recommendations for the (re)integration of minor returnees are valuable, especially since there is still little research available on the development and experiences of minor returnees.

**Supplementary Information:**

The online version contains supplementary material available at 10.1007/s40653-024-00656-z.

## Introduction

Growing up in a war situation with radicalized parents who are sentenced for their views after the war, causing children to reintegrate (temporarily) without parents into a society characterized by prejudice and different norms and values, is a potential threat to childrens' development. Dutch children of parents who joined the German Schutzstaffel (SS) or the Dutch National Socialist Movement (NSB) during World War II (WWII) grew up in this situation. This study primarily explores their experiences throughout childhood and adulthood, the implications of their experiences, and their coping responses, from their own perspective. It secondarily focuses on participants' recommendations for the successful reintegration of a new group of children growing up in a similar situation, i.e., minor returnees from the caliphate. Despite significant differences between their parents’ ideologies, the similarities in the circumstances that both groups of children grew up in are notable, which leads us to believe that the experiences of Dutch children of NSB and SS members can be helpful for minor returnees.

The NSB was a Dutch political party that collaborated with the Nazi regime (Orlow, 1999). The party emerged in 1931 during the Great Collapse (1929–1939), which was marked by massive unemployment (Pells & Romer, 2023). NSB members were feared by the vast majority of the Dutch population due to their active assistance to the Nazi regime. They for instance rounded up Jewish people, assisted in their deportation to Nazi destruction camps, and tracked down, detained, and executed people resisting the German occupiers (“The Holocaust,” 1990). After the war, the NSB was banned and members were brought to trial, detained (Brants, 2015), and despised for decades by the vast majority of Dutch society (Damsma, 2013). The exact number of Dutch children of NSB and SS members is unknown, but estimates range from 300,000 to 750,000 (Scheffel-Baars et al., 2004). Although much anecdotal (auto)biographical literature is available on these children, systematic studies of this population are scarce. Lindt (1993) conducted interviews with six children of NSB members and concluded that the threat of being associated with their parents was the root cause of the negative consequences experienced by these children. Lindt also identified several factors that contributed to successful coping with this background, including feeling accepted and understood, being approached neutrally, sharing experiences with peers, speaking out against parents, self-acceptance through knowledge and understanding of their background, self-determination, and acceptance of their parents.

Tames (2009) focused on the same population and examined the archives of welfare and governmental organizations that were founded for the identification and care of Dutch children of NSB and SS members between 1944 and 1960. She also interviewed adult Dutch children of NSB and SS members and studied their autobiographical materials. Tames concluded that for most of these children, the post-war period was characterized by the arrest of their parent(s), disruption of families, social isolation, secrecy about their origins, and a lack of consideration for their experiences. They were considered politically 'tainted' and therefore had to be 're-educated.' Many of the children felt they were forced to assimilate. Tames describes how the attention and care for these children gradually faded after their parents' release, and not referring to the war seemed to be the best way for the children and their families to live more or less normal lives. As a result, many of these children grew up with a strong fear of being exposed.

Finally, Aerts (2018) studied newspaper articles and conducted more than sixty interviews with adult children of Flemish collaborators during WWII. He argues that these children were severely stigmatized by society and neglected by the Belgian government. As a result, many of these children considered themselves victims in retrospect, not of their parents' actions but of a lack of vision and policy on the part of the Belgian government.

The primary aim of this study is to expand on this previous research by exploring childhood and adulthood experiences of the Dutch children of NSB and SS members, the impact these experiences had throughout their lives, and their coping responses both as children and as adults. The exploration of both childhood *and* adulthood experiences is new and contributes to a better understanding of the short-term and long-term needs of children growing up in a war situation and with radicalized parents. We investigate the following research questions related to this primary aim: (1) What were the experiences of Dutch children that resulted from their parents’ background as NSB and SS members during childhood and adulthood? (2) What implications did these experiences have for their development during childhood and adulthood? (3) How did they cope with these experiences during childhood and adulthood? 

The secondary aim of this study is to explore participants’ recommendations for the succesful (re)integration of minor returnees from the caliphate. Since the self-proclamation of the caliphate in June 2014, thousands of Western men and women have travelled to Syria and Iraq to help build the Islamic State. Nearly ten years later, many of the men have died or been detained, and many of their wives and children have ended up in Kurdish camps in northern Syria. Most of these women, some of them with children, have expressed their wish to return to their home countries. By spring 2024, a total of approximately 76 Dutch children had returned to the Netherlands. Since 2016, 35 children were reported to a Dutch embassy in Turkey by their parents after fleeing Syria. Since June 2020, a total of 41 children were repatriated from Kurdish camps as a result of a court ruling that impunity would be imminent for Dutch women in Kurdish camps if they were not given the opportunity to attend their criminal trials in the Netherlands. These children’s parents, suspected of affiliation with a terrorist group, are arrested and detained upon their return to the Netherlands. Only breastfed children under 9 months are allowed to remain with their mothers. Children over 9 months are initially placed in a care facility for a 3-month observation and stabilization period before being placed with their relatives? (if possible). After the parents are released from prison, the juvenile court decides whether the children can return to their parent(s). No figures are available on how many Dutch children are currently reunited with their parent(s). Around 140 children of Dutch descent remain in Syria, of whom approximately 25 in Kurdish camps, 75 in northwestern Syria and 40 elsewhere in Syria (Koninkrijksrelaties, 2024).

These minor returnees from the caliphate, like Dutch children of NSB and SS parents, grew up in a war situation with radicalized parents and had to (re)integrate into a society characterized by prejudice and different norms and values, often in the absence of their detained parents. Other similarities include the facts that both groups were taken abroad by their parents (Admin, 2020; Sandelowsky-Bosman & Liefaard, 2022), were in a relatively good materialistic position during the first period of the war (Reuters, 2017; Visser, 2022), and had to flee from approaching allied forces, resulting in the loss of their personal belongings (UNICEF, 2017; Visser, 2022). They both faced shelling and bombing (NOS, 1945; Gruiters, 2019) and several older children from both groups were sent to ideological education and/or combat training by their parents (Koninkrijksrelaties, 2024; Nationale Jeugdstorm, n.d.). Children from both groups were wounded or lost relatives due to the war (Avrotros, 2022; Koninkrijksrelaties, 2024). Both groups ended up in foreign camps with their mothers and siblings, where living conditions were sparse (DPG Media Privacy Gate, n.d.; UNICEF, 2019) and where most of them lost contact with their fathers (Ministerie van Buitenlandse Zaken en Koninkrijksrelaties, 2024; Stichting Werkgroep Herkenning, 2015b). Many children from both groups were born abroad and/or in these camps, which later caused legal problems upon their return to the Netherlands (Sandelowsky & Liefaard, 2019; Stichting Werkgroep Herkenning, 2015b). Parents from both groups were arrested and detained upon their return to the Netherlands, resulting in the separation of children from their primary attachment figures and causing them to be raised by others (Feenstra, 2022; NOS, 1945). Both groups grew up with their parents' extremist ideology, norms, and values, which differed from those of Dutch society and required them to adapt (Ministerie van Buitenlandse Zaken en Koninkrijksrelaties, 2023; Tames, 2009). Both groups were seen as alleged threats by Dutch society due to their parents' ideology (Stichting Werkgroep Herkenning, 2015c; Van der Heide & Geenen, 2017; Verzamelaccount, 2018). This is in line with a study from Capone (2017) that indicates that all children who grow up in extremist environments are perceived as a potential threat. Parents from both groups had to rebuild their lives from scratch after serving their detention (Feenstra, 2022; Tames, 2009), and children from both groups were not allowed to visit their detained parents regularly (Kouwenhoven & Van Der Poel, 2023; Stichting Werkgroep Herkenning, 2015a, b, c). For a complete overview of the similarities between both groups, see online Appendix A.

In addition to these similarities, there are also differences between the two groups of children such as the content of the ideological beliefs of their parents. The NSB adhered to a political right-wing extremist and ethnocentric ideology, pursued authoritarian, military supported political leadership and propagated nationalism, anti-Semitism and anti-communism. In contrast, ISIS adheres to a religious fundamentalist ideology, pursues a global theocratic state, led by a religious leader with religious, political, military and economic authority, which is underpinned by strict religious laws and jurisprudence (Moghadam, 2008).

However, despite the different ideological and demographic backgrounds of the childrens' parents, both groups of children were confronted with quite similar circumstances. Given these similarities, the secondary aim of this study was therefore to ask Dutch -now eldery- children of NSB and SS members for their recommendations regarding the (re)integration of minor returnees from the caliphate. Accordingly, the fourth research question is: (4) What can be learned from participants' experiences and recommendations on (re)integration of minor returnees?

## Methods

### Participants

Participants were members of Stichting Herkenning, a Dutch self-support group for individuals with at least one parent who was a member of the NSB or SS during WWII. The chairperson of the self-support group was informed of the study and invited members of the self-support group to participate in the interviews. This resulted in 17 participants (11 women [64.7%]), varying in age between 71–81 years old, with an average age of 76.14 years (*SD* = 3.19). Participants received a written explanation of the interview procedure and were asked whether they preferred a group or an individual interview, or had no preference. Four female participants indicated no preference. They were invited to a group interview, as all other participants preferred an individual interview. All participants provided written informed consent. The study procedures were approved by the faculty’s Ethical Review Board (BLINDED).

### Material

The semi-structured interview consisted of two parts. In the first part of the interview, participants were asked for recommendations on how to deal with minor returnees based on their own experiences of growing up in a war situation with radicalized parents (question 1 through 10). In the second part of the interview, participants were asked about their own experiences (question 11 through 21) in line with the primary aim of this study. Question 11 refers to the experiences of Dutch children of NSB and SS members as reported by Stichting Herkenning in their Compendium (2015). Initially, the order of the first and second part of the interview questions was reversed and in line with the primary and secondary aim of this study. However, the trial interview revealed that participants were so eager to talk about their experiences that hardly any time remained for interview questions about recommendations regarding the (re)integration of minor returnees. For this practical reason we reversed the two parts of interview questions.

To construct the interview, potentially relevant topics and sensitizing concepts—concepts that draw attention to important features of social interaction and provide guidelines for research (Gilgun, 2019)- were extracted and categorized from both grey literature (Cohen, 2020; Den Braber, 2014; Kooijmans, 2015; Van der Heijden, 2014) and academic literature (Aarts, 2018; Lindt, 1993; Tames, 2009). These sources were used to create a comprehensive topic list for the interviews (Baarda et al., 2013). Next, the first author (MM) formulated interview questions based on this topic list, and both the topic list and the interview questions were revised (i.e., de-duplicated, reordered and accentuated) by the research group (all authors). A trial version with nineteen interview questions was used in the first individual interview, which led to the addition of two more interview questions (Appendix B). Subsequently, a group interview was conducted to identify any potential missing topics or interview questions that could be incorporated into subsequent individual interviews. However, no new topics emerged from the group interview and no further modifications were deemed necessary. Therefore, it was possible to consider the data collected from the group and individual interviews as one body of data. Participants from the group interview and from the individual interviews were asked the exact same interview questions (Appendix B).

### Procedure

The participants were interviewed between November 2018 and February 2019. Of the thirteen individual interviews, ten took place at the participants' homes and three at an office of (BLINDED). Interviews had an approximate duration of three hours. All interviews were conducted by the first author (MM) and one of two co-interviewers (CD and MZ). All three interviewers were female, aged 45–60, educated in the same psychological discipline, and working as child psychologists at (BLINDED). All interviews were audio-recorded and transcribed verbatim.

Approximately one week after each interview, participants were telephoned by the first author (MM) to evaluate how they experienced the interview, whether they needed professional assistance, had missed any questions, or had perceived some questions as unnecessary. All participants indicated that they had experienced the interview as positive and responded negatively to the other questions. Any information derived from these phone calls was added to the verbatim transcripts of the interviews.

### Coding of the interviews

A codebook, presented in Appendix C, was developed for the coding process. The coding procedure is outlined in more detail below:

1. The first author (MM) and both co-interviewers (CD and MZ) independently read and analyzed the fourteen verbatim transcriptions of the group and individual interviews. They identified and compiled a list of codes derived from the interviews, which were categorized based on the four main interview topics: experiences, implications, coping responses, and recommendations for minor returnees. After analyzing the first ten verbatim interviews, no new codes emerged, indicating that saturation had been reached. Overall, a total of 185 codes were derived from the interviews (see Appendix C).
2. The first author (MM) proceeded to group the codes into themes, resulting in a total of 34 themes. For instance, codes related to recommendations regarding minor returnees were organized into themes targeting six different audiences: professionals/schools, society, governments, media, relatives and minor returnees themselves. Three themes were aligned with existing theoretical categorizations. That is, the theme of possible trigger factors for radicalization was divided into factors at the personal, group, and social level, in line with Doosje et al. (2016). The theme of attachment problems was further categorized as inhibited and disinhibited attachment problems, as described by Gleason et al. (Gleason et al., 2011). The coping theme was aligned with the scales of the Utrecht Coping List, a widely used questionnaire that measures coping styles (Schreurs & Willige, 1993).
3. All authors (BD, AE, BJ, HB, MM) then reviewed and de-duplicated the list of codes and themes.
4. To increase the interrater reliability of the scoring procedure of the codes, one randomly selected interview was scored by two authors (MM and BJ) using Atlas.ti version 8 (https://atlasti.com). Scores were compared and differences were resolved through discussion. Both authors next scored codes of one additional, randomly selected interview. For this interview, the number of agreements had increased to an adequate level (> 70%, as recommended by Salkind, 2010).
5. Finally, the remaining 12 interviews were scored by the first author (MM) using Atlas.ti version 8. The scored codes can be found in Appendix C.

### Data analysis

In accordance with Baarda (2013), the research material was further analyzed as follows. Using Atlas.ti, the frequency (S) of each of the 185 codes in the joint interviews was counted. Additionally, the number of interviews (NI) in which each code was mentioned was determined to measure its dispersion within the sample. For the first three main interview topics (experiences, implications, and coping), only codes with NI-scores of nine or higher were presented. This criterion was applied to highlight the key experiences, implications, and coping responses while avoiding overemphasizing codes that occurred frequently but in only a few interviews. As 41 of the 185 codes met the NI cut-off of nine or higher, this threshold struck a good balance between sparsity and representativeness of the results. Other NI cut-off results are available from the first author upon request.

While the codes of the three main interview topics emerged spontaneously in the interviews, this was different for the codes of the fourth interview topic (recommendations), where participants were asked directly about their recommendations for minor returnees. As a result, the recommendations recurred in all the interviews, causing the NI-value to lose meaning. It was therefore decided to judge the importance of the recommendations by the number of times each specific recommendation was mentioned in the interviews as a whole and to present the two most frequently mentioned codes for each theme. For the theme “Recommendations for schools and other professionals” we present the four most frequently mentioned codes because this theme consisted of a relatively large number of codes (eighteen).

## Results

Participants' key experiences, implications and coping responses (all NI ≥ 9) and their key recommendations for minor returnees (highest S-scores) are described below. Illustrating quotes are marked with a superscript, referring to a specific participant, allowing the reader to see that quotes come from different participants.

### Results Related to Interview Topic 1: Experiences

Table 1 shows an overview of codes related to the key experiences (NI ≥ 9) mentioned by participants. The codes fell in the themes living conditions after the war, social experiences, high impact experiences, and possible trigger factors for radicalization, the latter at a personal level as well as at a societal level.

**Table 1:** Codes and illustrative quotes regarding participants’ experiences

Theme	Code	NI
Living conditions after the war M(NI) = 9.13	Had various caregivers	14
	Family had lost all possessions	12
	Sparse living conditions	11
	Had parent(s) in internment camp(s)	10
	Lived in a (network) foster family	9
Social experiences as a child M(NI): 8.80	Felt accepted	13
	Felt left out / isolated	11
Social experiences as an adult M(NI): 7.25	Felt accepted	10
	Felt left out / isolated	9
High impact experiences within the family of origin M(NI): 6.00	Suffered from secrecy within the family	12
	Loss of parent(s) or sibling(s)	10
Possible trigger factors for radicalization at a social level M(NI): 6.00	Has experienced social prejudice, stigmatization	13
Possible trigger factors for radicalization at a personal level M(NI): 5.00	Has met a radical person	10

Only codes reported in ≥ 9 interviews are shown. *NI*, number of interviews that a code was mentioned in; *M(NI)* average of NI’s of all codes within a theme

It is noteworthy that participants predominantly described the challenging living conditions after the war, rather than during the war itself. As one participant explained, “For us, the war only started when it ended for others”. Furthermore, many participants reported that their *parents were placed in internment camps after the war (NI:10)* and that their *family lost all their possessions* due to confiscation *(NI:12*), leaving them with *sparse living conditions (NI:11).* One participant said: “My mother had been arrested and my 4 siblings took care of me. My 8-year-old brother went out begging for food. It’s a miracle I survived.”^(12)^ In cases where their mothers were not detained, they had to work long hours to earn an income, leaving the participants *under the care of (network) foster families (NI:*9). The frequent relocations to different (network) foster families or shelters meant that participants *often had multiple caretakers (NI:14).*

Regarding their *social experiences*, participants expressed that they constantly *felt left out and isolated, both during childhood (NI:11) and adulthood (NI:9)*. One participant recalled: “Every time I left the house, I was bullied.”^(13)^ Many participants referred to a speech by Queen Wilhelmina after World War II, in which she declared that there was no place in Dutch society for traitors. This statement immediately made participants feel excluded and continues to be deeply hurtful even today. Many participants pointed out the enduring stigmatization that they still face today, more than 75 years later. Or as one participant told: “In the retirement home where I live, I received a note under my door saying: “You nasty, nasty NSB child.”^(11)^ Although participants also mentioned positive social experiences where they *felt accepted by others both during childhood (NI:13) and adulthood (NI:10),* a closer examination of the associated quotes reveals that during childhood, these instances of acceptance were limited to specific situations, such as interactions with compassionate relatives or children whose parents were unaware of the NSB or SS background of the participants' parents. One participant described: “I had four friends, which was special because I was never welcome at other children’s homes. These four girls were new to our neighbourhood, so their parents didn’t know my parents.”.^(2)^

When examining *the key high-impact experiences* described by participants, it is notable that all these experiences occurred *during childhood* and *within their families*. These experiences included *suffering due to secrecy within the family (NI:12)* and *the loss of parent(s) or sibling(s) (NI:10)* due to the war.

Among the key experiences mentioned by participants, *contact with a radical person (NI:10)* was identified as a possible trigger factor for radicalization (Doosje et al., 2016). A participant described: “After the war I once took the metro with my father. Very proudly he said: “And here I removed so many (read: Jewish people) from their homes.” My father remained proud of his deeds for a long time.”^(9)^. Additionally, *experiencing social prejudice and stigmatization (NI:13)* was recognized as a possible trigger factor for radicalization at the societal level (Doosje et al., 2016). However, the president of the self-help group states that none of the Dutch children of former NSB or SS members have ever been implicated in extremist motives. She explains, "We all made great efforts to assimilate and demonstrate that we were not like our parents".

### Results Related to Interview Topic 2: Implications

Table 2 shows an overview of the key implications (NI ≥ 9) of participants' experiences during childhood and adulthood. They concern their relationships with relatives, their moral development, their development of identity, and negative core beliefs regarding themselves, in both childhood and adulthood.

**Table 2:** Implications of background related experiences for participants’ development and adult life: themes and underlying codes

Theme	Code	NI
Family relationships: from (a) family member(s) towards the participant M(NI): 9.50	Experienced support from a relative	14
	Experienced a relative as estranged	12
	Experienced parent(s) as emotionally unavailable	10
	Experienced resentment from a relative	10
Negative core beliefs about themselves M(NI): 9.33	Something is wrong with me	10
	I should not complain or ask for attention	10
Emotions experienced as a child M(NI): 7.14	Joy	11
	Fear	9
	Love	9
Family relationships: from interviewee towards family member(s) M(NI): 6.83	Conflicting feelings of (dis)loyalty towards relative	14
	Feels /felt tension within the family	10
Emotions experienced as an adult M(NI): 6.29	Grief	9
	Shame / guilt	9
Moral development M(NI): 6.00	Needed external information to form his/her own judgement	11
Identity development M(NI): 5.17	Not knowing your own father or own history	9
	Difficult or late development of personal identity	9

Only codes reported in ≥ 9 interviews are shown. *NI*, the number of interviews the code was mentioned in; *M(NI)*, average of NI’s of all codes within a theme

Participants’ experiences had impact on their *emotions during both childhood and adulthood.* During childhood they often felt *fear (NI:9).* Participants mostly described this as *a general feeling of fear*, *a constant vigilant state of being* (of note, this increased vigilance could still be observed in several interviews). Only in specific situations where they experienced protection, they felt *joy (NI:11)* and *love (NI:9).* One participant explained: “I was always welcome with my grandmother. She spoiled me. She made me feel special.”^(9)^ Perhaps one would have expected that participants' experiences would also have led to feelings of anger because they were seen as an alleged threat to society, just like minor returnees from the caliphate, due to the extremist ideology of their parents. However, feelings of anger were mentioned in only three interviews, and in all cases, participants' anger was directed at their fathers, not at Dutch society. During adulthood many of them did experience *shame/guilt (NI:9)* and *grief (NI:9)* due to their parents’ background. Or as one participant mentioned: “I’m still not done crying. I’m emotionally incontinent.”^(5)^ Participants described these emotions as a reason to start therapy or to join a self-help group.

Participants’ experiences also caused *negative core beliefs about themselves*, especially during childhood. Key core beliefs included *“I shouldn’t complain or ask for attention”(NI:10)* and *“Something is wrong with me” (NI:10).* One participant described: “The parents of children I played with were so unkind to me, so I often looked in the mirror and thought: “What’s wrong with me?”^(9)^ Several participants mentioned that during childhood they could feel a tension in their family, but that due to family secrecy they could not understand what caused this tension. As a result, they looked for causes within themselves. One participant described: “I was so insecure. I still am. I never feel good enough.”.^(9)^

Participants’ background experiences also *impacted their relationships with relatives.* Due to family secrecy, participants often *felt tensions in their relationships with family members (NI:10)*. All participants reported *conflicting feelings of (dis)loyalty towards their parent(s) (NI:14).* They disapproved of their parents' choices and actions related to the NSB or SS, but also loved them for the parents they were to them. Several participants mentioned that they felt a lifelong pressure from society to condemn their parent(s). Although all participants *felt support or protection from a relative (NI:14*) during childhood, mainly from those who cared for them during their parents' absence, they also often *felt resentment from relatives (N:10),* who took out their frustrations on the children instead of their (arrested, absent) parents. One participant mentioned: “My grandmother didn’t like us going to live with her. We had to eat in the kitchen and sleep in a corner of the attic. She hired a carriage and drove us through the destroyed, bombed city saying: “Look at what your friends did.”^(14)^ After the reunion with their parents, many participants *experienced their parent(s) as estranged (NI:12)* and *emotionally unavailable (NI:10)* as they were primarily concerned with their own problems and rebuilding their lives. One participant mentioned: “I had lived with my aunt for a long time after the war. When my mother finally came to pick me up, I greeted her saying: “Good day madam.” I didn’t recognize her anymore.”.^(5)^

A key implication for participants’ *moral development* was their *need for external information (NI:11)* to figure out that their parents' political beliefs, norms and values differed from the prevailing norms and values in society. Several participants mentioned that history classes in school, which were very unnuanced and reinforced feelings of shame and guilt and the fear of being exposed, as well as the need to remain silent, played an important role in this process. During adulthood they were in need of external information about what their parents actually had or had not engaged in during the war. One participant explained: “In history class, I learned about the war. The teacher told a completely different story than my parents did. No, I had no doubts. My parents were wrong. That’s how it felt, immediately.”.^(5)^

Participants’ experiences also had implications for their *identity development.* Many mentioned that they experienced *a difficult, delayed development of their personal identity (NI:9)*. Or as one participant stated: “I don’t know whether I’ve already started developing it.”^(7)^ This had two reasons. First, several participants *grew up not knowing who their fathers were (NI:9)*. They were untraceable after the war, or were kept hidden or lied about by their mothers. One participant illustrated: “My mother had misled me. For years I had believed that my father was executed. But that wasn’t my father. My real father turned out to be a true war criminal.”^(5)^ A second reason was the ongoing genetically related question: “If my parents were war criminals and I have their genes, then who or what am I?”.

### Results Related to Interview Topic 3: Coping Responses

Table 3 shows participants’ key coping responses (NI ≥ 9). Coping responses were divided into three themes, corresponding to widely acknowledged types of coping (Schreurs & Willige, 1993): active coping, avoidant coping, and reappraisal.

**Table 3:** Participants’ coping responses: themes and underlying codes

Theme	Code	NI
Active response M(NI): 9.00	Searched for information about parents	14
	Making personal history discussable	10
Avoiding response M(NI): 6.33	Keeping the past a secret	11
	Socially desirable/ overly adapted behavior	9
Reappraisal M(NI): 5.88	Placing parents’ choices in a broader context	13
	Attributing positive traits to the parent(s)	10

Only codes reported in ≥ 9 interviews are shown. *NI* the number of interviews the code was mentioned in; *M(NI)*, average of NI’s of all codes within a theme

The two coping responses that participants considered most effective were *active coping strategies*: *searching for information about their parent(s) (NI:14),* especially in the National Archive, and *making personal history discussable (NI:10).* Many participants remarked that it was more helpful to share their experiences with peers than with professionals because peers provided them with more recognition and acknowledgment than professionals.

Another key coping response was *keeping the past a secret (NI:11)*, a type of *avoidant coping*, which participants considered harmful in the long run. Almost all participants indicated that after the war, they initially chose not to talk about their past due to their parents' and their own desire to leave that past behind and start a new life. They managed to maintain secrecy for years, but this eventually led to serious emotional or physical complaints. Participants indicated that, in retrospect, they should have been open about their personal history much earlier. One participant mentioned: “I had never told my wife that my father had been an NSB-member during the war. I didn’t think it was necessary. At some point, she found out and started to blame me for keeping it a secret.”^(3)^ Another avoidant coping response mentioned in many interviews was *overly adaptive behavior (NI:9)*. Participants described this behavior as a result of years of social exclusion, judgment, and stigma. They often maintained this coping response throughout their lives. Or as one participant said: “I tried so hard. I thought if I just act nice and do exactly what they want me to do, then I can probably participate.”.^(1)^

Most participants demonstrated the deployment of the third type of coping, *reappraisal,* by *placing their parents' choice to join the NSB or SS in a broader context (NI:13)* For example, they mentioned factors such as the fear of the Antichrist, the worldwide economic crises, and high unemployment rates before WWII as reasons that led their parents to join the NSB or SS. Additionally, participants *attributed positive traits to their parents (NI:10)* during the interviews without in any way trying to diminish or deny their parents’ responsibility for their political choices and actions.

### Results Related to Interview Topic 4: Recommendations

Finally, participants were asked for their recommendations concerning minor returnees from the caliphate for professionals, schools, society at large, governments and politicians, media, parent(s) and other family members, and minor returnees themselves. Table 4 shows an overview of the recommendations mentioned (see Table 4).

**Table 4:** Participants’ recommendations concerning minor returnees

Theme	Code	S
Recommendations for professionals and schools M(S): 16.11	Talk with minor returnees	34
	Be honest and transparent with these children	26
	Approach them as individuals and with empathy	24
	Stimulate the relationship with their parent(s)	18
Recommendations for society M(S): 15.25	Do not condemn minor returnees	42
	Man is not his behaviour or choices	11
Recommendations for governments and politicians M(S): 10.00	Do not separate the children from the mothers	10
	Offer the mother(s) housing, don’t let them have to live-in with family	3
Recommendations for media M(S): 7.75	Respect minor returnees’ privacy	15
	Offer society a nuanced perception	10
Recommendations for the parent(s) and family of minor returnees M(S): 6.83	Be transparent and honest about parents’ choices	18
	Offer love and boundaries	7
Recommendations for minor returnees M(S): 5.40	No need to judge your parent(s)	10
	You are not your parent(s)	5

Codes with the four highest S-scores (S) are presented for the theme “Recommendations for professionals and schools” and codes with the two highest S-scores are presented for the remaining themes. *S*, frequency of the code in the joint interviews; *M(S)*, average frequency of all codes of a theme

First, the recommendations of participants emphasized that *minor returnees should be approached without prejudice and in a non-judgmental manner*. Children should in no way be held responsible for the choices and actions of their parents or for their parents' adherence to certain ideological groups. Minor returnees should be treated with empathy and as individuals. Several participants expressed the sincere hope that, for the sake of the children, media and school curricula should provide a nuanced perspective on the background of minor returnees and thereby help pave the way for the succesfull (re)integration of minor returnees (see for example code “Do not condemn minor returnees” under theme “Recommendations for society” in Table 4).

The second feature that participants' recommendations had in common was the need to *be transparent and honest with minor returnees about their past and to avoid secrecy* (see for example code “Be transparent and honest about parents’ choices” under theme “Recommendations for the parent(s) and family of minor returnees” in Table 4). Participants emphasized that children not only have the right to know their own past, but also need this knowledge to gain a better understanding of other people's reactions and to avoid attributing the cause of these reactions to themselves. Knowledge of their past can also help them avoid being overwhelmed or surprised when questions arise about their birthplace or other aspects of their past.

The third feature that emerged from the participants' recommendations is *the importance of the bond between minor returnees and their parents*. Participants stated that minor returnees should receive explicit permission to remain loyal to their parents, not to their choices or actions. They should not feel any social pressure to distance themselves from their parents. As one participant stated, "Each child remains a child of his or her parents, whether we like it or not." Relatedly, participants emphasized that minor returnees should preferably not be separated from their mothers upon return (as is currently the case in many, if not most, of their home countries), because the mother is the child's main attachment figure. Participants argued that, in the interest of these children, their mothers should receive support to rebuild their lives. Two illustrative quotes were: “Everyone is afraid that these children will follow the same path as their parents. But look at Dutch children of NSB and SS members. There were so many more of them, none of them posed a danger to society.”^(12)^ and “At the end of the war, the Nazi regime transported children of Dutch women and German soldiers from Dutch maternity hospitals to Germany, without their mothers. The children ended up in German children's homes. After the war, the Dutch government refused to repatriate these children. But eventually, after much ado, the children returned in the 1950s. Much suffering could have been prevented by having them return immediately. I’m afraid that history will repeat itself.”.^(12)^

### Tentative developments in experiences, implications, and coping responses across the lifespan

**Fig. 1:**
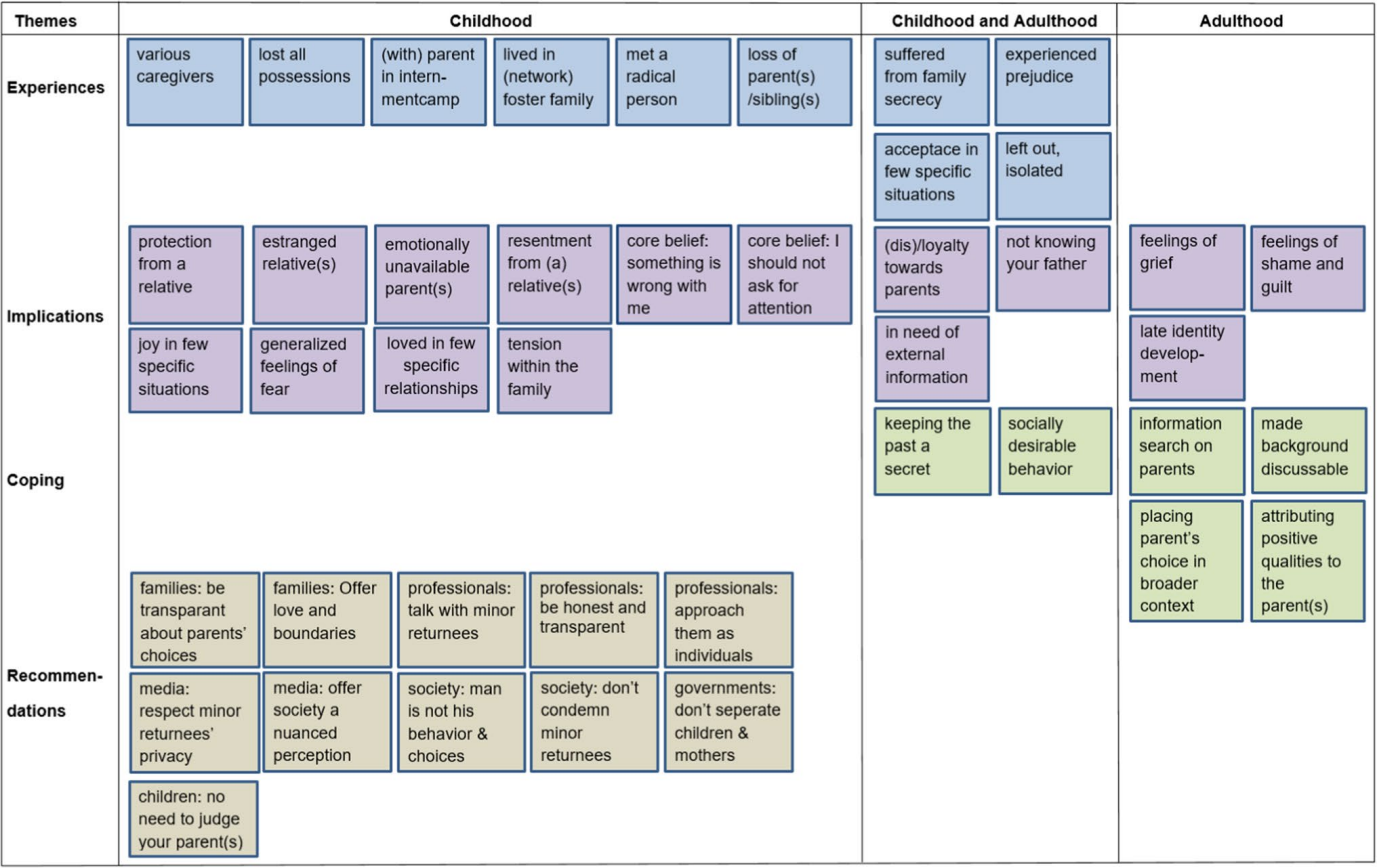
Participants’ key experiences, implications, coping responses and recommendations

Although the data do not allow to draw conclusions on cause and effect, we have attempted to display the sequence of experiences, implications, coping strategies and recommendations in Fig. 1. All key codes (NI Codes that were mentioned in 9 or more interviews were included in Fig. 1. Experiences, implications and coping strategies are divided into "childhood", "adulthood" or "both" and give an indication of the life course of the participants.

Examining Fig. 1, we see that most of the participants' key childhood experiences were related to being separated from their parents, who were arrested and detained right after the war. We also see that the *suffering from family secrecy* regarding their parents' NSB or SS membership started during childhood and apparently continued into their adult lives. This is also the case for their feelings of *being excluded* by the vast majority of people.

Looking at *the implications of participants' experiences*, we see a wide range of consequences starting in participants' childhood and continuing throughout the rest of their lives. None of *the coping responses* that participants mentioned applied specifically to childhood, and we also see that active coping responses were not applied until adulthood.

When we look at the *participants' recommendations regarding minor returnees*, we see that they all relate to the childhood of minor returnees. Furthermore, participants believe that for the (re)integration of minor returnees, an active role is needed not only from individuals directly involved with the children but also from groups at a meso-level, such as the media, politicians, and society.

## Discussion

The primary aim of this study was to gain a better understanding of the experiences, their implications, and the coping responses of Dutch children of NSB and SS members, and to explore whether these were specifically related to their childhood, adulthood, or both.

The secondary aim of this study was to generate recommendations for the succesful (re)integration of minor returnees from the caliphate.

Regarding the primary aim of this study, several conclusions can be drawn. First, the interviews indicate that it is probable that the detention of participants’ parents has led to anxiety and distress, specifically during participants' childhood. This aligns with research that has found that children with detained parent(s) experience increased levels of stress (Zayas et al., 2015). The lack of emotional availability of the parent(s) to reassure the child after the separation, and the lack of information about why the parent is absent, can exacerbate the child’s distress and lead to trauma (Murray et al., 2012). We also observed this in our interviews with the Dutch children of NSB and SS members, who reported the arrest and detainment of their parents as a key experience during childhood and the emotional unavailability of parents as an important influence on their childhood.

Second, we found that participants reported stigmatization as a key experience both during childhood and adulthood. Possibly related with this, they reported that they felt isolated and unaccepted throughout their lives and only felt accepted in specific relationships with either compassionate family members or friends who were unaware of their parents' background. These results are in line with a study on long-term effects of stigmatization among German children born from intimate relationships between occupying allied forces’ soldiers and local German women after WWII (Kaiser, 2022). These children (and their mothers) also experienced severe stigmatization within their own families, immediate social circles, and public institutions throughout their childhood and adolescence, and reported withdrawal and avoidance as frequent coping behaviours.

Third, we found that experiences of family secrecy which began in childhood and sometimes persisted well into adulthood, severely impacted participants’ relationships with their parents, family members, and their self-perception throughout their lives. In line with this idea, earlier research shows that by 6 years of age, children understand that social relationships may involve obligations to keep each other's secrets, and that failing to do so can harm these relationships (Liberman, 2020). This may explain why many participants dared not ask their parents about the tension they felt in the family. Not knowing where the feelings of tension in the family came from caused participants to doubt and blame themselves, resulting in negative self-perceptions that persisted into their adult lives. This is in line with Barnwell (2019), who states that family secrecy often stems from good intentions such as wanting to protect family members from perceived social risk and sanctions, but as a downside has the effect that underlying trauma persists as long as the family secret endures.

Fourth, we found that growing up with radicalized parents and sometimes lifelong family secrecy about the parents' background, may have resulted in conflicting feelings of loyalty and disloyalty toward these parents throughout our participants' lives, as well as in feelings of sadness, shame and guilt that often lasted well into adulthood. Due to the concealment of their parents' background, participants were often painfully confronted for the first time at school –particularly during history classes- with the fact that their parents had been supportive of the Nazi regime and how this was judged by the majority of the Dutch population. Another key implication of the family secrecy seems to be that, as adults, many participants started to search for information on their parents' activities during the war in the Dutch National Archives. Participants reported that their ongoing search for new information on their parents, along with the often unknown identities of German SS fathers (another reported key influence on participants’ childhood and adulthood), caused problems that may have contributed to yet another key implication of their experiences: a delayed identity development, often voiced in the question, "If these were my parents, who am I?" As described by Stets and Burke (2003), personal identities are maintained through a feedback control process, in which one’s perceived identity is compared to a societal standard. Any discrepancy between the two is resolved by adjusting behaviour, perception, or identity standards. This theory explains why identity development becomes problematic and delayed for individuals who throughout their lives persistently search for and find new information that deviates from the norm and from their desired social identity.

Fifth, participants reported that a general feeling of fear during childhood developed into a continuous state of hypervigilance during adulthood. Causes of hypervigilance indeed include trauma and stigma (Kimble et al., 2023) as experienced by many our participants.

Sixth, we found that in adulthood, our participants eventually developed adequate coping responses for dealing with their problems, such as seeking professional help, joining a peer group, sharing their experiences, and seeking information about their parents. In contrast, they reported only two key coping responses in childhood, i.e., overly adaptive behaviour and concealment, which were clearly perceived to be ineffective. Of note, our participants did not report the use of common positive coping strategies during their childhood, such as direct problem-solving, seeking social support or finding distraction in playing (Nijboer, 2007). Perhaps participants in the current study did not mention such strategies because they did not recollect them, but it may also be the case that such strategies were not employed.

In addition to strengthening the findings of Lindt (1993), Tames (2009), and Aerts (2018), this study contributes to new knowledge regarding the experiences, the implications of these experiences and the coping responses of Dutch children of NSB and SS members in specific life stages including their childhood, adulthood or both. This contributes to a better understanding of the short-term and long-term needs of Dutch children of NSB and SS members who grew up in a war situation with radicalized parents and had to reintegrate in a society with prejudice and different norms and values.

Regarding the secondary aim of this study, participants provided numerous recommendations for the (re)integration of minor returnees from the caliphate.

Combining the experiences of the Dutch children of NSB and SS members, their recommendations, and relevant literature, we suggest the following recommendations for the successfull (re)integration of minor returnees from the caliphate. First, in cases where minor returnees are separated from their parents upon return, we recommend to prioritize keeping siblings together (Barnea et al., 2023), to explain to minor returnees why their detained parents are absent, to facilitate frequent visiting rights, to provide support to the parent(s) after their release from prison to ensure optimal emotional availability to their children (Murray et al., 2012), and to repair any damage done in the attachment relationship between minor returnees and their parents due to their separation upon return.

Second, it is advisable to acknowledge and facilitate the importance of minor returnees’ (family) networks, as children of NSB and SS members found solace, affection, and happiness in their relationships with understanding and accepting relatives. However, given the painful experiences that some participants also reported within their families, we recommend that family members involved with the minor returnees receive psycho-education on the consequences of concealing the past and the identity of the fathers for the minor returnees. It is also recommended to support the family members involved with the minor returnees in recognizing signs and symptoms of psychological trauma and adopting a trauma-sensitive approach (Lambert & Lynch, 2016).

Third, we believe the answer to the question of effective coping is more nuanced than the choice between revealing or concealing the past, since the participants in our study reported that they suffered from the fact that their parents' NSB or SS membership was not talked about within the family, but at the same time also reported that after the war they only felt accepted by children whose parents did not know about the (former) NSB or SS membership of participants’ parents. Particpants’ recommendation to teach minor returnees to talk openly about their background from an early age also contrasts with Tames' (2009) observation that, in retrospect, not discussing the war seemed to be the best way for children of NSB families to lead relatively normal lives after the war. Similarly, Kuterovac et al. (Franc & Kuterovac-Jagodić, 1998) found that childrens' active coping response of talking about the war was ineffective and associated with high levels of intrusive psychological symptoms.

We therefore recommend a compromise between disclosure and concealment that is tailored to the needs of a specific child. We recommend appointing confidants among people directly involved with the minor returnees on a day-to-day basis—outside the family context—such as a teacher or sports coach, for example, who should be aware of a child's past and to whom a child can turn with questions or a need of support. A study by Denov (2010) supports participants’ recommendation that peer groups can be supportive for children who have experienced a war. We therefore also recommend the establishment of peer groups for minor returnees where, in line with the experiences of the participants of this study, they can find recognition for their shared experiences.

Fourth, with respect to minor returnees who may have received ideological education or combat training in the caliphate, we believe it is important for family members and confidants of the minor returnees not to conceal the extremist context in which they grew up. As noted by Brando and Echeverry (2022), treating potentially trained children as mere victims may incapacitate them and deprive them of control over their own choice to consciously distance themselves from past behavior.

Fifth, we emphasize the importance of paying attention to the identity development of minor returnees. It is crucial not to conceal their background and their fathers for them. Providing them with age-appropriate information about their parents' choices and lives in Syria or Iraq at a young age, can equip them with a narrative when asked about their background and allow them to explore their roots (Woodham et al., 2017) before encountering such inquiries unprepared at school or elsewhere. As such, we recommend creating a life story with each minor returnee with the assistance of a parent or person directly involved in the child's daily life (Hein & Schlattmann, 2021), to give new meaning to experiences and allow possible trauma to be addressed and treated. However, it is quite possible that minor returnees who were born in Syria and lived in the multilingual population of Kurdish camps may not speak and/or understand the Dutch language well upon their return to the Netherlands. This may be a barrier in profiting from the education and assistance offered to them upon their return to the Netherlands.

Sixth, we observe that both children of NSB and SS members, as well as minor returnees, have experienced stigmatization, which arises from an intergroup process (Link & Phelan, 2006). To combat stigmatization of minor returnees, it would be helpful if media provide society with accurate, objective, non-stigmatizing information and balanced narratives regarding minor returnees and their parents and respect their privacy. Governments should support social organizations, educational institutions, and municipalities in developing policies and practices that promote inclusion.

Seventh, we recommend the establishment of long-term monitoring and support systems to assess the well-being and development of minor returnees, considering the potential long-term impact of their experiences (Dye, 2018). We also believe further research is needed to evaluate the effects of these and possible other recommendations and interventions.

There are several limitations that must be considered when interpreting our findings. First, our participants are not representative of the total population of children of NSB and SS members. They all, to some extent, experienced problems related to their parents’ background, actively went looking for help, joined a self-help group and chose to disclose their past. Participants’ assertion that talking about the past is a more effective coping response than keeping the past a secret, may for instance have emerged because of this selection bias. Second, the participants' experiences took place a long time ago. Without in any way wishing to detract from the participants' memory capacities or the meaning that the recounted experiences had and still have for them, research also shows that memories are not static and may be subject to forgetting or reconstructive processes (Bernstein & Loftus, 2008). Third, four participants were interviewed as a group. Group dynamics may have stimulated talkativeness or instead the restraint to openly share the burden of their personal background. Fourth, the decision to only describe the most frequently mentioned codes, does not imply that codes that were less frequently mentioned may not be important on the individual level.

Despite these limitations, we believe that the findings of this study contribute to a better understanding of the experiences of Dutch children of NSB or SS members, the implications of these experiences, and their coping responses during childhood, adulthood, or both. We also believe that the participants' recommendations, potentially make an important contribution to the successful (re)integration of minor returnees from the caliphate.

## Supplementary Information

Below is the link to the electronic supplementary material.

Supplementary file1 (DOCX 27 KB)

Supplementary file2 (DOCX 37 KB)

Supplementary file3 (DOCX 45 KB)

## Data Availability

This study was not pre-registered. Anonymous research data are available upon reasonable request. The data are not publicly available due to privacy and ethical restrictions.
