# Supplementary material for: Growing up with Radicalized Parents: The Experiences of Dutch Children of NSB and SS members During and After World War II
Source: J Child Adolesc Trauma. 2024 Sep 13;18(1):35–48. doi: 10.1007/s40653-024-00656-z (PMC11910471; doi:10.1007/s40653-024-00656-z)
Supplement: Supplementary file 1 — Supplementary file1 (DOCX 27 KB) [file 40653_2024_656_MOESM1_ESM.docx]

| **Children of (a) Dutch NSB or SS member(s) in WWII** | **Children of (a) Dutch national(s) who joined a Jihadi group in the last decade in Syria or Iraq and have returned to the Netherlands** |
| --- | --- |
| Many of these children were taken from their familiar environment in the Netherlands (on so-called Dolle Dinsdag, when the Allies approached the Dutch border) to Germany by their parents *Source:*[*https://www.historischnieuwsblad.nl/het-lot-van-naar-duitsland-gevluchte-nsbers/*](https://www.historischnieuwsblad.nl/het-lot-van-naar-duitsland-gevluchte-nsbers/)*)* | Many of these children were taken from their familiar environment in the Netherlands to Syria or Iraq by their parents  *Source:*  [*https://repository.wodc.nl/bitstream/handle/20.500.12832/3219/JV202203_artikel6.pdf?sequence=8&isAllowed=y*](https://repository.wodc.nl/bitstream/handle/20.500.12832/3219/JV202203_artikel6.pdf?sequence=8&isAllowed=y) |
| From a materialistic point of view, these children initially led *relatively* good lives during the war  *Source:* [*https://historiek.net/nsb-nationaal-socialistische-beweging-tweede-wereldoorlog/129548*](https://historiek.net/nsb-nationaal-socialistische-beweging-tweede-wereldoorlog/129548) | From a materialistic point op view, these children initially still led *relatively* good lives in Raqqa during the war  [*https://www.jpost.com/middle-east/islamic-state-cleared-from-syrias-raqqa-507658*](https://www.jpost.com/middle-east/islamic-state-cleared-from-syrias-raqqa-507658) |
| These children had to flee from the Netherlands with their families , leaving most of their personal belongings behind  *Source:* [*https://historiek.net/nsb-nationaal-socialistische-beweging-tweede-wereldoorlog/129548/*](https://historiek.net/nsb-nationaal-socialistische-beweging-tweede-wereldoorlog/129548/) | These children had to flee from Raqqa with their families, leaving most of their personal belongings behind  *Source:*[*https://www.unicef.nl/pers/2017-06-09-40-000-kinderen-vast-in-de-vuurlinie-bij-raqqa-terwijl-gevechten-verergeren/*](https://www.unicef.nl/pers/2017-06-09-40-000-kinderen-vast-in-de-vuurlinie-bij-raqqa-terwijl-gevechten-verergeren/) |
| These children experienced shelling and bombing  *Source:*[*https://nos.nl/75jaarbevrijding/bericht/2300592-ruim-dertig-doden-bij-beschieting-trein-met-vluchtende-nsb-ers*](https://nos.nl/75jaarbevrijding/bericht/2300592-ruim-dertig-doden-bij-beschieting-trein-met-vluchtende-nsb-ers) | These children experienced shelling and bombing  *Source:*[*https://jangruiters.wordpress.com/2019/03/11/de-overwinningsnederlaag-in-baghuz/*](https://jangruiters.wordpress.com/2019/03/11/de-overwinningsnederlaag-in-baghuz/)*)* |
| Older children could be signed up by their parents for special education in Germany, designed to strengthen the ideology they adhered to  *Source:*[*https://www.oorlogsbronnen.nl/thema/Nationale%20Jeugdstorm*](https://www.oorlogsbronnen.nl/thema/Nationale%20Jeugdstorm)*)* | Older children could be signed up by their parents for special education in Iraq or Syria, designed to strengthen the ideology they adhered to  *Source:*  *<https://www.aivd.nl › publicaties › 2017/04/06)>* |
| Older children could also be signed up by their parents for combat training. Some of these children lost their lives in combat  *Sources:* [*https://www.avrotros.nl/actualiteit/item/hitlers-kindsoldaten/*](https://www.avrotros.nl/actualiteit/item/hitlers-kindsoldaten/) | Older children could also be signed up by their parents for combat training. Some of these children lost their lives in combat  *Source:*  *<https://www.aivd.nl › publicaties › 2017/04/06)>* |
| At the end of the war these children were held in German camps together with their mothers  *Source:* [*https://www.parool.nl/nieuws/veel-nsb-ers-in-kamp*](https://www.parool.nl/nieuws/veel-nsb-ers-in-kamp) | At the end of the war these children were held in Kurdish camps together with their mothers  *Source:* [*https://www.unicef.nl/pers/2019-05-22-unicef-landen-moeten-kinderen-in-syrische-kampen-meer-beschermen*](https://www.unicef.nl/pers/2019-05-22-unicef-landen-moeten-kinderen-in-syrische-kampen-meer-beschermen) |
| When they were in the German camps, most of these children lost contact with their fathers *Source:*[*https://www.werkgroepherkenning.nl/kenniscentrum/geschiedenis-2/de-periode-1948-1963/*](https://www.werkgroepherkenning.nl/kenniscentrum/geschiedenis-2/de-periode-1948-1963/) | When they were in the Kurdish camps, most of these children lost contact with their fathers  *Source:*[*https://www.aivd.nl/onderwerpen/terrorisme/uitreizigers-en-terugkeerders*](https://www.aivd.nl/onderwerpen/terrorisme/uitreizigers-en-terugkeerders) |
| Living conditions in the German camps -where many of these children stayed before returning to the Netherlands- were sparse  *Source:* [*https://nos.nl/75j/2318955*](https://nos.nl/75j/2318955)*)* | Living conditions in the Kurdish camps -where many of these children stayed before returning to the Netherlands- were sparse  *Source:* [*https://www.unicef.nl/pers/2019-05-22-unicef-landen-moeten-kinderen-in-syrische-kampen-meer-beschermen*](https://www.unicef.nl/pers/2019-05-22-unicef-landen-moeten-kinderen-in-syrische-kampen-meer-beschermen)*)* |
| Many of these children were born in Germany (or) in camps for Dutch NSB- or SS-members, which after the war caused problems and questions after their return to the Netherlands  *Source:*[*https://www.werkgroepherkenning.nl/voor-wie/kinderen-van-duitse-militairen*](https://www.werkgroepherkenning.nl/voor-wie/kinderen-van-duitse-militairen) | Many of these children were born Syria or Iraq (or) in Kurdish camps, which caused problems and questions after their return to the Netherlands  *Source:* [*https://njcm.nl/wpcontent/uploads/2020/04/4.NTM-44-3_Sandelowsky-BosmanLiefaard_De-verantwoordelijkheid-van-Nederland-voor-kinderen-met-een-Nederlandse-link-in-voormalige-IS-strijdgebieden_pdf-g.pdf*](https://njcm.nl/wpcontent/uploads/2020/04/4.NTM-44-3_Sandelowsky-BosmanLiefaard_De-verantwoordelijkheid-van-Nederland-voor-kinderen-met-een-Nederlandse-link-in-voormalige-IS-strijdgebieden_pdf-g.pdf) |
| After their return to the Netherlands the families had to rebuild their lives from scratch  *Source:*[*https://www.academia.edu/44231216/Besmette_jeugd_Kinderen_van_NSBers_na_de_oorlog*](https://www.academia.edu/44231216/Besmette_jeugd_Kinderen_van_NSBers_na_de_oorlog) | After their return to the Netherlands the families had to rebuild their lives from scratch  *Source:* [*https://nos.nl/artikel/2450635-wat-staat-de-twaalf-opgehaalde-is-vrouwen-uit-Syrie-te-wachten*](https://nos.nl/artikel/2450635-wat-staat-de-twaalf-opgehaalde-is-vrouwen-uit-Syrie-te-wachten) |
| After returning to the Netherlands, most of the parents were arrested and detained, which led to the children being separated from their parents and having to be cared for by someone else  *Source:*[*https://nos.nl/75jaarbevrijding/bericht/2341135-nsb-kind-voor-ons-begon-de-oorlog-na-de-bevrijding*](https://nos.nl/75jaarbevrijding/bericht/2341135-nsb-kind-voor-ons-begon-de-oorlog-na-de-bevrijding) | After returning to the Netherlands, most of the parents were arrested and detained, which led to the children being separated from their parents and having to be cared for by someone else  *Source:* [*https://nos.nl/artikel/2450635-wat-staat-de-twaalf-opgehaalde-is-vrouwen-uit-Syrie-te-wachten*](https://nos.nl/artikel/2450635-wat-staat-de-twaalf-opgehaalde-is-vrouwen-uit-Syrie-te-wachten) |
| These children grew up with the ideology, norms and values of their radicalized parent(s), which differed from those of the Dutch society in which the children had to (re)integrate. This appealed to the adaptive skills of these children  *Tames, I. (2009). Besmette jeugd: de kinderen van NSB’ers na de oorlog. Balans.* | These children grew up with the ideology, norms and values of their radicalized parent(s), which differed from those of the Dutch society in which the children had to (re)integrate. This appeals to the adaptive skills of these children  *Source:*[*https://www.aivd.nl/onderwerpen/terrorisme/uitreizigers-en-terugkeerders*](https://www.aivd.nl/onderwerpen/terrorisme/uitreizigers-en-terugkeerders) |
| They were seen by society as a potential threat because of the ideology of their parents  *Source:*[*https://www.werkgroepherkenning.nl/interview-de-juffrouw-zei-jij-hoort-niet-meer-bij-nederland/*](https://www.werkgroepherkenning.nl/interview-de-juffrouw-zei-jij-hoort-niet-meer-bij-nederland/) | They were seen by society as a potential threat because of the ideology of their parents  *Source:*[*https://www.ewmagazine.nl/nederland/opinie/2018/05/terughalen-is-kinderen-veiligheidsprobleem-616041/*](https://www.ewmagazine.nl/nederland/opinie/2018/05/terughalen-is-kinderen-veiligheidsprobleem-616041/) |

| These children were only occasionally allowed to visit their detained parent(s)he children could rarely visit their detained parent(s)  *Source:*[*https://www.werkgroepherkenning.nl/voor-wie/het-laatste-taboe-in-nederland-nsb-kinderen/kinderkamp/*](https://www.werkgroepherkenning.nl/voor-wie/het-laatste-taboe-in-nederland-nsb-kinderen/kinderkamp/) | These children are only occasionally allowed to visit their detained parent(s)  *Source:* [*https://kinderenteruguitkalifaat.nl/wp-content/uploads/2023/04/20230408-nrc-bericht-uit-de-zwolse-vrouwenvleugel.pdf*](https://kinderenteruguitkalifaat.nl/wp-content/uploads/2023/04/20230408-nrc-bericht-uit-de-zwolse-vrouwenvleugel.pdf) |
| --- | --- |
| Up until their return to the Netherlands and their parents' imprisonment, these children generally had their mothers as the most important attachment figure in their lives. Upon their return to the Netherlands they were seperated from their mothers, who were arrested and detained *Source:*[*https://historiek.net/nsb-nationaal-socialistische-beweging-tweede-wereldoorlog/129548/*](https://historiek.net/nsb-nationaal-socialistische-beweging-tweede-wereldoorlog/129548/) | Up until their return to the Netherlands and their parents' imprisonment, these children generally had their mothers as the most important attachment figure in their lives. Upon their return to the Netherlands they were seperated from their mothers, who were arrested and detained *Sources:*[*https://dekanttekening.nl/nieuws/ngo-maant-nederland-teruggekeerde-is-moeders-niet-scheiden-van-kinderen/*](https://dekanttekening.nl/nieuws/ngo-maant-nederland-teruggekeerde-is-moeders-niet-scheiden-van-kinderen/) *&* |
