# Supplementary material for: Growing up with Radicalized Parents: The Experiences of Dutch Children of NSB and SS members During and After World War II
Source: J Child Adolesc Trauma. 2024 Sep 13;18(1):35–48. doi: 10.1007/s40653-024-00656-z (PMC11910471; doi:10.1007/s40653-024-00656-z)
Supplement: Supplementary file 2 — Supplementary file2 (DOCX 37 KB) [file 40653_2024_656_MOESM2_ESM.docx]

**Questions for the interviews with Dutch children of NSB- and SS-members**

* These questions were added after the trial interview

1. What can we learn from your childhood experiences, when it comes to Dutch and Flemish children returning from Syria or Iraq?

2. Given your own experiences, what do you think should be arranged for Dutch and Flemish children who come (back) from Syria or Iraq to the Netherlands or Belgium?

3. Based on the experiences from your past, what will give the Dutch and Flemish children returning from Syria and Iraq, the resilience to develop positively after returning to the Netherlands or Belgium?

4. Based on your experience, what do you consider most relevant to ask Dutch and Flemish children returning from Syria and Iraq and their parents about:

- The period before the trip to Syria / Iraq (if parents took them on their journey)?
- The period in "the caliphate"?
- The period in Syria or Iraq after the fall of "the caliphate"?
- The procedure of return to the Netherlands or Flanders?
- The period after returning to the Netherlands or Flanders?

5. What would you advise Dutch and Flemish children returning from Syria and Iraq?

6. What would you like to advise the parents of these children?

7. What would you advise the educators/family members of these children?

8. What would you advise the professionals that help these children?

9. Do you think that these children can pose a safety risk to Dutch or Belgian society? Would you please explain your answer?

10.^*^ What do you think our research on minor returnees should focus on? What should we investigate when it concerns these children?

11. The Compendium of Stichting Werkgroep Herkenning (Chapter 5) describes experiences that children of parents who made a 'wrong' choice may have experienced. We would like to include these experiences, where relevant, in the study of minors returning from Syria or Iraq. In order not to overlook important, relevant experiences, we would like to ask you if, based on your own experiences, you are still missing experiences/topics in the below summary from the Compendium (ASK PARTICIPANT TO READ AND COMMENT ON LIST A-D):

a) Concerning the family of origin:

- Keeping the past a secret within the family
- Feeling, as a child, that there is a secret in the family, but not knowing what this secret is
- Lower socio-economic status and financial problems of the family after the war
- Indoctrination within the family
- Being registered for the 'Jeugdstorm' by the parents, not by own choice
- To be confronted with fear and uncertainty from parents
- Father's departure for an unknown time to Germany or Russia
- Mothers who fled to Germany with children while fathers had to stay behind in the Netherlands
- Loss of family roots
- Having to go to a German school without speaking the language
- Confiscation of the family property after the war
- Having witnessed violence and assault on parental arrest
- A stay in internment and transit camps, under conditions with poor food, bad hygiene and diseases
- Evidence of alienation between family members after family reunification
- Little emotional availability of parents who are dealing with their own problems
- Marriage dissolution
- After the arrest of parents, being cared for by family or in a children's home without parents
- Being cared for outside the family with or without brothers and sisters
- No reunification with the original family, but a permanent residence outside the

family

- Discussing and exposing the political choices of parents after the war
- No financial resources available for further education of the children
- Tension and frustration in the family, due to difficulty in finding work (at an appropriate level)
- Loyalty conflicts for the family member who is the first to seek help outside the family

Are there any experiences concerning your family of origin that you miss in the above list, which you consider relevant to include in the study of children who have lived in a former ISIS area?

b) Concerning social contacts with others:

- Being avoided or bullied by classmates or children on the street

- Neighbours or relatives who keep a distance from your family of origin

- Being received by family members while feeling that you are "a burden" to them

- The feeling of being accountable for the political choice of your parent(s)

- Not being admitted to associations

- Inquiry from neighbors about the functioning of your family of origin, by the Political Offender Supervision Foundation

- Isolate yourself from others as a family or as a child

- Adjusting excessively to others, wanting to do well all the time

- Wanting to show that you make different political choices than your parents

Did you have other experiences in social contacts with others that you miss in the above list and that you consider relevant to include in the study of children who have lived in a former ISIS area?

c) Concerning war experiences:

- Having experienced aircraft bombing

- Being forced to re-educate

- Long after the war, once again being confronted with the experiences one wants to leave behind, e.g., through renewed media attention for WWII

- Having experienced the war differently than most of the Dutch citizens

- Having experienced the war differently from from what is taught in history lessons at school

Are there any war experiences that you miss in the above list and which you consider relevant to include in the research on children who have lived in a former ISIS area?

d) Concerning grandchildren (if applicable):

- Maintaining the family secret in order not to endanger the relationship between

grandchildren and grandparents

- Transferring your own fears and experiences as a child to your own children

- Being confronted with the coping responses you developed as a child of parents who made the “wrong” choice

Are there any experiences regarding grandchildren that you miss in the above list and which you consider relevant to include in the study of children who have lived in a former ISIS area?

12. What is your date of birth?

13. Do you have concrete memories of the Second World War? To what extent have you experienced that having or not having concrete memories of the war, can be of help in processing your wartime history?

14. Have you been a member of the ‘Jeugdstorm’ or another National Socialist Youth Movement? If so, what positive and / or negative memories do you have of this?

15. a) When and what made you realize for the first time that you are a child of

parents who made a 'wrong' choice? What did this mean for you then?

b) Did this change the relationship with your parents and if so, how?

c) From what age onwards would you have liked to talk to others about the political orientation of your parents/family and if so, with whom?

d) Were you able to discuss this with family members? How did you like that?

16. What impact has your background had on:

a) Your school performance? (learning disabilities?)

b) Your emotions?

c) Your contacts and relationships with others?

d) Your physical health?

e) Your personality?

f) Your mental health? (diagnoses?)

g) Your behavior?

h) Your self-image?

i) Other aspects of your development?

17. a) What did you find most difficult about the fact that your parents were seen by

others as ‘wrong’ during the war?

b) How did you deal with this as a child?

c) What gave you support?

d) How did your parents deal with this?

18. What made you feel safe, if so, after the war?

19. Has the fact that you were a child of parents who made a 'wrong' choice during the war, affected how you later related to:

1. Dutch society, and if so how?
2. Politics and your political preference, and if so how?
3. Do you see a crucial role for parents in this?

20.^*^ At which moment(s) in your life, if so, did you get stuck and how did you deal with this?

21. What help, if so, did you need as a child after the war?
