# Supplementary material for: Growing up with Radicalized Parents: The Experiences of Dutch Children of NSB and SS members During and After World War II
Source: J Child Adolesc Trauma. 2024 Sep 13;18(1):35–48. doi: 10.1007/s40653-024-00656-z (PMC11910471; doi:10.1007/s40653-024-00656-z)
Supplement: Supplementary file 3 — Supplementary file3 (DOCX 45 KB) [file 40653_2024_656_MOESM3_ESM.docx]

| **Interview topic** | **Theme** | **Code** | **S** | **%** | **NI** |
| --- | --- | --- | --- | --- | --- |
| **1.1 What background-related experiences did the participants mention?** | - - 1. Living conditions due during the war   Σ = 73  N = 6  Average NI = 5,00 | 1.1.1.1 had to flee  1.1.1.2 sparse living conditions: poverty. cold. little to eat  1.1.1.3 was separated from the parent(s)  1.1.1.4 had a family member who attended the ‘Jeugdstorm’  1.1.1.5 lived-in with other people together with his/her parent (s)  1.1.1.6 had various caretakers | 10  16  22  16  4  5 | 13,7 %  21,9%  30,1%  21,9%  5,5%  6,8% | 6  5  7  7  2  3 |
|  | - - 1. Living conditions after the war   Σ = 215  N = 8  Average NI = 9,13 | 1.1.2.1 stayed in a detainment camp with parent(s)  1.1.2.2 family had lost all possessions  1.1.2.3 had parent(s) in detainment camp(s)  1.1.2.4 did not know his / her father  1.1.2.5 sparse living conditions  1.1.2.6 lived in a (network) foster family  1.1.2.7 lived with other people together with his/her parent (s)  1.1.2.8 had various educators | 9  34  39  24  31  35  18  25 | 4,2%  15,8%  18,1%  11,2%  14,4%  16,3%  8,4%  11,6% | 4  12  10  5  11  9  8  14 |
|  | 1.1.3 Social experiences as a child  Σ = 112  N = 5  Average NI = 8,80 | 1.1.3.1 felt left out / isolated  1.1.3.2 felt bullied / violated / humiliated  1.1.3.3 didn’t understand other people’s reaction to him/her  1.1.3.4 felt accepted  1.1.3.5 experienced loss of contact or break up with friends | 32  23  20  33  4 | 28,6%  20,5%  17,9%  29,5%  3,6% | 11  8  8  13  4 |
|  | 1.1.4 Social experiences as an adult  Σ = 49  N = 4  Average NI = 7,25 | 1.1.4.1 felt left out / isolated  1.1.4.2 felt bullied, violated or humiliated  1.1.4.3 felt accepted  1.1.4.4 experienced loss of contact / break up with friends / partner (s) | 16  6  17  10 | 32,7%  12,2%  34,7%  20,4% | 9  4  10  6 |
|  | 1.1.5 Experiences with high impact within the family of origin  Σ = 185  N = 8  Average NI = 6,00 | 1.1.5.1 poverty  1.1.5.2 mental violence  1.1.5.3 physical violence  1.1.5.4 sexual violence  1.1.5.5 loss of parent (s) or sibling(s)  1.1.5.6 family members with trauma / serious mental health problems  1.1.5.7 suffered from secrecy within the family  1.1.5.8 was lied to by the parent (s) | 15  10  11  1  28  6  77  37 | 8,1%  5,4%  5,9%  0,5%  15,1%  3,2%  41,6%  20,0% | 4  5  5  1  10  4  12  7 |
|  | 1.1.6 Experiences with high impact outside the family of origin  Σ = 43  N = 6  Average NI = 4,67 | 1.1.6.1 poverty  1.1.6.2 mental violence (also harassment. exclusion. stigmatization)  1.1.6.3 physical violence  1.1.6.4 sexual violence  1.1.6.5 shelling and bombing  1.1.6.6 visit to / stay in (a) detainment camp(s) or prison(s) | 4  10  4  8  11  6 | 9,3%  23,3%  9,3%  18,6%  25,6%  14,0% | 4  6  4  5  6  3 |
|  | 1.1.7 Possible trigger factors for radicalization at a personal  level  Σ = 35  N = 4  Average NI = 5,00 | 1.1.7.1 has faced death  1.1.7.2 had early school leaving / job loss  1.1.7.3 has met a radical person  1.1.7.4 confronted with propaganda | 1  10  24  0 | 2,9%  28,6%  68,6%  0% | 1  9  10  0 |
|  | 1.1.8 Possible trigger factors for radicalization at a group level  Σ = 0  N = 3  Average NI = 0,00 | 1.1.8.1 was a member of Jeugdstorm (youth association of the NSB)  1.1.8.2 broke ties with the group to which he / she previously belonged  1.1.8.3 heard a call from the radical group to join or to take violent action | 0  0  0 | 0%  0%  0% | 0  0  0 |
|  | 1.1.9 Possible trigger factors for radicalization at a societal level  Σ = 80  N = 4  Average NI = 6,00 | 1.1.9.1 has experienced social prejudice, stigmatization  1.1.9.2 had a collision with authorities  1.1.9.3 felt hurt by statements of authorities  1.1.9.4 experienced military actions or attacks against a social group | 58  6  10  6 | 72,5%  7,5%  12,5%  7,5% | 13  2  7  2 |
| - 1. **What were the implications of participants’ background–related experiences for their development and adult life?** | 1.2.1. Signs of inhibited type of attachment problems  Σ = 45  N = 3  Average NI = 5,33 | 1.2.1.1 is overly vigilant in social situations  1.2.1.2 shows a withdrawn, invisible attitude  1.2.1.3 had/has little confidence in others | 18  4  23 | 40,0%  8,9%  51,1% | 7  1  8 |
|  | 1.2.2 Signs of disinhibited type of attachment problems  Σ = 17  N = 5  Average NI = 1,60 | 1.2.2.1 shows excessive need for attention  1.2.2.2 is a friend to all people; too much physical proximity  1.2.2.3 problems to maintain relationships  1.2.2.4 has difficulty with delay in satisfaction of needs.  1.2.2.5 is busy, Impulsive, unfocused | 4  1  9  0  3 | 23,5%  5,9%  52,9%  0%  17,6% | 2  1  3  0  2 |
|  | 1.2.3 Emotions experienced as a child  Σ = 116  N = 7  Average NI = 7,14 | 1.2.3.1 love  1.2.3.2 fear  1.2.3.3 joy  1.2.3.4 anger  1.2.3.5 grief  1.2.3.6 amazement/astonishment  1.2.3.7 shame / guilt | 18  31  23  4  19  4  17 | 15,5%  26,7%  19,8%  3,4%  16,4%  3,4%  14,7% | 9  9  11  3  8  3  7 |
|  | 1.2.4 Emotions experienced as an adult  Σ = 91  N = 7  Average NI = 6,29 | 1.2.4.1 love  1.2.4.2 fear  1.2.4.3 joy  1.2.4.4 anger  1.2.4.5 grief  1.2.4.6 surprise / amazement  1.2.4.7 shame or guilt | 2  16  17  10  20  6  20 | 2,2%  17,6%  18,7%  11,0%  22,0%  6,6%  22,0% | 1  8  8  5  9  4  9 |
|  | 1.2.5 Functioning at school  Σ = 21  N = 4  Average NI = 2,50 | 1.2.5.1 fear of failure, insecure  1.2.5.2 concentration problems  1.2.5.3 learning difficulties  1.2.5.4 had a language barrier after a stay in Germany | 10  6  5  0 | 47,6%  28,6%  23,8%  0% | 4  3  3  0 |
|  | 1.2.6 Negative core beliefs about themselves  Σ = 87  N = 3  Average NI = 9,33 | 1.2.6.1 something is wrong with me  1.2.6.2 I am accountable  1.2.6.3 I should not complain or ask for attention | 45  21  21 | 51,7%  24,1%  24,1% | 10  8  10 |
|  | 1.2.7 Experienced family relationships from the participant  towards family member(s)  Σ = 125  N = 6  Average NI = 6,83 | 1.2.7.1 take care of parent (s). parentification  1.2.7.2 self isolation towards other family members  1.2.7.3 was disappointed in another family member / relative  1.2.7.4 was angry / rebellious with a family member / relative  1.2.7.5 feelings of loyalty and/or disloyalty towards a relative  1.2.7.6 felt/feels tension within the family | 12  0  6  13  57  37 | 9,6%  0%  4,8%  10,4%  45,6%  29,6% | 7  0  3  7  14  10 |
|  | 1.2.8 Experienced family relationships from family member(s)  towards the participant  Σ = 158  N = 6  Average NI = 9,50 | 1.2.8.1 felt support / protection from a relative  1.2.8.2 has experienced a relative as estranged  1.2.8.3 has experienced parent(s) as emotionally unavailable  1.2.8.4 has experienced resentment from a relative  1.2.8.5 felt overly protected by a relative  1.2.8.6 has experienced indoctrination attempts by another family member | 50  32  31  14  10  21 | 31,6%  20,3%  19,6%  8,9%  6,3%  13% | 14  12  10  10  5  6 |
|  | 1.2.9 Poor health conditions as a child  Σ = 21  N = 6  Average NI = 3,17 | 1.2.9.1 physical diseases due to the war  1.2.9.2 mental disorder(s) due to the war  1.2.9.3 physical injuries due to the war  1.2.9.4 malnutrition due to the war  1.2.9.5 persistent stress / tension  1.2.9.6 psychosomatic complaints due to the war | 9  2  0  4  5  1 | 42,9%  9,5%  0%  19,0%  23,8%  4,8% | 7  2  0  4  5  1 |
|  | 1.2.10 Poor health conditions as an adult  Σ = 29  N = 4  Average NI = 3,50 | 1.2.10.1 persistent stress / tension  1.2.10.2 permanent physical injuries from the war  1.2.10.3 mental disorders  1.2.10.4 psycho somatic complaints (for example symptoms of paralysis) | 17  0  7  5 | 58,6%  0%  24,1%  17,2% | 8  0  3  3 |
|  | 1.2.11 Moral development  Σ = 63  N = 6  Average NI = 6,00 | 1.2.11.1 difficulty with prevailing values  1.2.11.2 difficulty with prevailing norms  1.2.11.3 a young child initially has no own moral judgment, independent from its parent(s)  1.2.11.4 needed external info to form his/her own judgment  1.2.11.5 feeling free to develop your own judgment depends on the degree of (dis)loyalty  one experiences towards his/ her parent(s)  1.2.11.6 wanting to be (extra) good as a counter-reaction on ‘wrong’ choices of parent(s) | 2  2  11  26  13  9 | 3,2%  3,2%  17,5%  41,3%  20,6%  14,3% | 2  2  6  11  8  7 |
|  | 1.2.12 Identity  Σ = 62  N = 6  Average NI = 5,17 | 1.2.12.1 not being able to separate yourself from your parent(s)  1.2.12.2 difficult / late development of identity  1.2.12.3 not knowing your own father and own history  1.2.12.4 being seen as a perpetrator  1.2.12.5 not using your family’s name  1.2.12.6 job as an identity | 11  15  21  4  7  4 | 17,7%  24,2%  33,9%  6,5%  11,3%  6,5% | 5  9  9  4  3  1 |
| **1.3 What coping responses do/did participants use?** | 1.3.1 Active coping response  Σ = 127  N = 4  Average NI = 9,00 | 1.3.1.1 making personal history discussable  1.3.1.2 searched for professional support /therapy  1.3.1.3 searched for information about parents  1.3.1.4 converted their own experiences into useful experiences | 46  13  57  11 | 36,2%  10,2%  44,9%  8,7% | 10  8  14  4 |
|  | 1.3.2 Palliative coping response  Σ = 18  N = 5  Average NI = 2,20 | 1.3.2.1 reading  1.3.2.2 taking care of a pet  1.3.2.3 meditation  1.3.2.4 fantasizing, magical thinking  1.3.2.5 use of alcohol, drugs. | 3  0  2  12  1 | 16,7%  0%  11,1%  66,7%  5,6% | 2  0  2  6  1 |
|  | 1.3.3 Avoiding coping response  Σ = 167  N = 9  Average NI = 6,33 | 1.3.3.1 hide  1.3.3.2 keeping the past a secret  1.3.3.3 shut itself off  1.3.3.4 pretend nothing happened  1.3.3.5 keep politically aloof  1.3.3.6 change / no longer use of the family’s name  1.3.3.7 keep a distance from their parent(s)  1.3.3.8 postponement of judgment  1.3.3.9 socially desirable/ adapted behavior | 21  77  13  24  7  7  2  4  12 | 12,6%  46,1%  7,8%  14,4%  4,2%  4,2%  1,2%  2,4%  7,2% | 8  11  7  7  7  4  1  3  9 |
|  | 1.3.4 Seeking social support  Σ = 28  N = 2  Average NI = 6,00 | 1.3.4.1 find a confidant  1.3.4.2 joined a peer group | 4  24 | 14,3%  85,8% | 4  8 |
|  | 1.3.5 Passive reaction pattern  Σ = 34  N = 4  Average NI = 3,50 | 1.3.5.1 endure patiently  1.3.5.2 worrying  1.3.5.3 feeling powerless  1.3.5.4 become entirely absorbed by the personal past | 8  1  17  8 | 23,5%  2,9%  50,0%  23,5% | 3  1  6  4 |
|  | 1.3.6 Seeking possibilities for expressing emotions  Σ = 21  N = 2  Average NI = 5,00 | 1.3.6.1 without words: drawing, painting, sculpturing, etc.  1.3.6.2 with words: writing (a book or poems) | 4  17 | 19,0%  81,0% | 3  7 |
|  | 1.3.7 Comforting thought  Σ = 174  N = 8  Average NI = 5,88 | 1.3.7.1 consider yourself separate from your parent(s)  1.3.7.2 see benefits of your past  1.3.7.3 having prepared a story in response to questions  1.3.7.4 see a new beginning; new opportunity  1.3.7.5 placing parent's choice in a broader context  1.3.7.6 the need to experience control  1.3.7.7 separate the behavior/ choices of the parent(s) from the parent(s) as a person  1.3.7.8 attributing positive traits to the parent(s) | 11  2  3  2  73  21  17  45 | 6,3%  1,1%  1,7%  1,1%  42,0%  12,1%  9,8%  25,9% | 5  1  1  2  13  7  8  10 |
| **2. 1 What recommendations do participants have concerning minor**  **returnees?** | 2.1.1 Recommendations for minor returnees  Σ = 27  N = 5  Average NI = 2,80 | 2.1.1.1 no need to judge your own parent  2.1.1.2 asking for help is always allowed  2.1.1.3 you are allowed to say no  2.1.1.4 you are not your parent(s)  2.1.1.5 additional recommendations | 10  1  1  5  10 | 37,0%  3,7%  3,7%  18,5%  37,0% | 2  1  1  4  6 |
|  | 2.1.2 Recommendations for the parents and families of minor  returnees  Σ = 41  N = 6  Average NI = 4,33 | 2.1.2.1 prepare a story the child can tell  2.1.2.2 be transparent and honest about parents’ choices  2.1.2.3 offer love and boundaries  2.1.2.4 integrate for the sake of the children  2.1.2.5 check for nightmares  2.1.2.6 additional recommendations | 4  18  7  6  1  5 | 9,8%  43,9%  17,1%  14,6%  2,4%  12,2% | 3  8  7  3  1  4 |
|  | 2.1.3 Recommendations for society  Σ = 61  N = 4  Average NI = 4,75 | 2.1.3.1 man is not his behavior or choices  2.1.3.2 do not condemn minor returnees  2.1.3.3 asking for help is always allowed  2.1.3.4 additional recommendations | 11  42  1  7 | 18,0%  68,9%  1,6%  11,5% | 3  11  1  4 |
|  | 2.1.4 Recommendations for the media  Σ = 31  N = 4  Average NI = 3,50 | 2.1.4.1 offer society a nuanced perception  2.1.4.2 man is not his behavior or choices  2.1.4.3 respect minor returnees’ privacy  2.1.4.4 additional recommendations | 10  5  15  1 | 32,3%  16,1%  48,4%  3,2% | 6  3  4  1 |
|  | 2.1.5 Recommendations for professional and schools  Σ = 290  N = 18  Average NI = 5,33 | 2.1.5.1 offer special attention in education  2.1.5.2 teach common standards and values  2.1.5.3 quickly teach them to speak Dutch  2.1.5.4 do not have opinions about these children and parent(s)  2.1.5.5 offer them play therapy  2.1.5.6 observe, follow the child, do not impose  2.1.5.7 place them within their families  2.1.5.8 receive them in a family context  2.1.5.9 guide their caregivers/teachers  2.1.5.10 approach them as individuals and with empathy  2.1.5.11 teach parents to talk to their child  2.1.5.12 be honest and transparent with these children  2.1.5.13 offer anonymity  2.1.5.14 stimulate relationship with the parent(s)  2.1.5.15 filing for later  2.1.5.16 stability and continuity  2.1.5.17 talk with minor returnees  2.1.5.18 additional recommendations | 2  7  11  14  8  10  14  10  15  24  9  26  2  18  12  6  34  68 | 0,7%  2,4%  3,8%  4,8%  2,8%  3,4%  4,8%  3,4%  5,2%  8,3%  3,1%  9,0%  0,7%  6,2%  4,1%  2,1%  11,7%  23,4% | 1  1  2  6  4  5  7  7  5  7  2  9  2  8  4  4  9  13 |
|  | 2.1.6 Recommendations for governments and politicians  Σ = 30  N = 3  Average NI = 5,67 | 2.1.6.1 do not separate the children from the mothers  2.1.6.2 offer the mothers housing quickly, don’t let them have to live-in with family  2.1.6.3 additional recommendations | 10  3  17 | 33,3%  10,0%  56,7% | 5  3  9 |

*Σ = frequency of theme codes in the fourteen interviews.*

*N = the number of different codes of a theme.*

*S= frequency of a single code in the fourteen interviews*

*NI= the number of interviews the single code was scored in.*
